# Neurosurgical apps for iPhone, iPod Touch, iPad and Android

**DOI:** 10.4103/2152-7806.74148

**Published:** 2010-12-22

**Authors:** Pieter L. Kubben

**Affiliations:** Department of Neurosurgery, Maastricht University Medical Center, The Netherlands

There is an abundance of useful content available in the App Store and Android Market for your mobile device, including medical content. *Surgical Neurology International* gladly supports the delivery of neurosurgical content on mobile devices with two active projects.

## SNI MOBILE

A few months ago, we released a web application for the journal that offered you a layout optimized for small screens for accessing our articles, posts, forum entries and videos. Nevertheless, the web application had the disadvantage that it reloaded its homepage every time you accessed it. This is due to the nature of web applications, but made browsing the content a little cumbersome.

**Figure 1:**
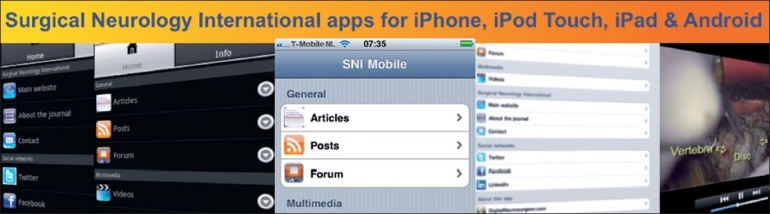
Screenshots of the SNI Mobile app

For that reason, we created native apps for iPhone, iPod Touch, iPad and Android [Figure 1], and they are now available as free downloads from the App Store and Android Market, respectively. They offer the same functionality, but faster and with a better user experience on these devices. Users of other devices can visit our homepage to get an optimized small screen layout for reading the articles. More instructions are available on our website from the “Mobile” section. This replaces the former “Neurosurgery 2.0” section, to ensure more intuitive navigation on the site. The links to our social networks have been replaced and are now accessible as icons from the menu bar itself.

## NEUROMIND

**Figure 2:**
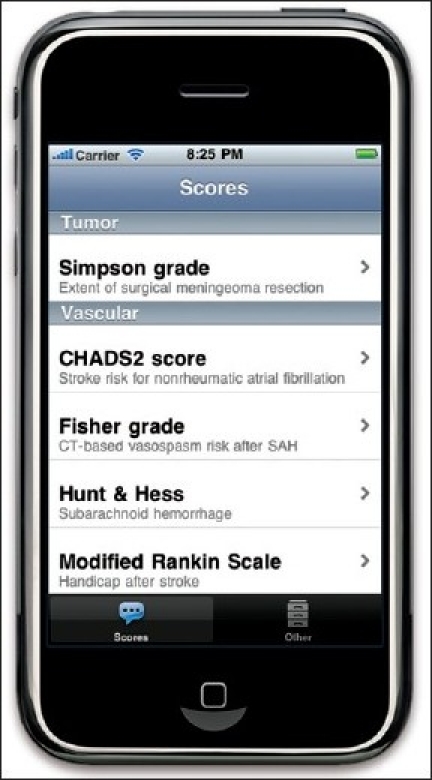
Preview of the new NeuroMind layout

The second project we are collaborating on is NeuroMind. Within six months after its release, it became the #1 ranked neurosurgical app in the App Store with over 30,000 downloads. It has been mentioned in several magazines and weblogs, for example the widely cited “Top 10 Free Medical iPhone apps” on iMedicalApps. com. From user evaluations, we learned that residents and neurosurgeons value the “Scores” the most for use in daily practice, followed by some anatomical illustrations for explanation to patients and students. Therefore, this part will be improved in the next major update, that will run on iPhone, iPod Touch, iPad and Android as well. An illustration of the new layout is visible in Figure 2. A first version for Android is already available in the Android Market, and the major upgrade is expected within two-three months. A web-based version is being developed to access the content from desktop computers and other mobile devices.

## FURTHER DEVELOPMENT

For more information on the progress and development of both apps, you can follow the Posts on our website. For technical details, you can visit the weblog on “DigitalNeurosurgeon.com”. Any requests for additional content can be sent to me by E-mail. We are looking forward to collaborating with our readers on expanding the mobile apps.

**Pieter Kubben**
Information Technology Editor,
Surgical Neurology International

